# Renal Angiomyolipoma: The Good, the Bad, and the Ugly

**DOI:** 10.5334/jbsr.1536

**Published:** 2018-04-20

**Authors:** Nicolas Vos, Raymond Oyen

**Affiliations:** 1 University Hospitals Leuven, BE

**Keywords:** angiomyolipoma, renal, tuberous sclerosis complex, imaging, therapy

## Abstract

Angiomyolipomas (AMLs) are the most common benign renal tumours. Most of these neoplasms are found incidentally on imaging. However, symptomatic presentation does exist.

Renal AMLs are typically composed of smooth muscle, blood vessels, and adipose tissue. Because of the abundant fat tissue, they give a characteristic appearance on imaging and are therefore easily diagnosed. However, sometimes they contain too little fat to be detected. This increases the difficulty in differentiating them from renal cell carcinoma (RCC).

Management of AML is based on clinical presentation and should be individualized for every patient. Treatment modalities range from active surveillance to more invasive approaches.

## Introduction

Angiomyolipomas (AMLs) are the most frequent benign renal tumour, with a prevalence varying between 0.2% and 0.6% and a strong female predilection. They occur as sporadic, isolated entities in 80% of cases. The remaining 20% of AMLs develop in association with tuberosclerosis complex (TSC) or pulmonary lymphangioleiomyomatosis (LAM) [1,2].

AMLs are considered as a heterogeneous group of neoplasms. Many types display different pathology, radiological features, and clinical behaviour, although they all consist of variable proportions of the same three elements: smooth muscle, blood vessels, and adipose tissue [2,3].

Imaging plays a central role in the diagnosis and management of renal AMLs. The detection of adipose tissue is the fundamental diagnostic criterion of a classic AML [2]. However, a minority of AMLs lack visually detectable fat on imaging, making it harder to distinguish from renal cell carcinoma (RCC). Therefore, accurate preoperative diagnosis of renal AMLs is critical to prevent unnecessary nephrectomies and preserve renal functions [4].

The purpose of this review is to provide a radiological classification of renal AML that contributes to the understanding and diagnosis of the different types and the appropriate therapeutic management and follow-up.

## Classifications

### Clinical Classification

About 80% of AMLs present as isolated entities, most commonly manifesting in middle-aged women. They tend to be single and small and rarely progress to cause significant morbidity [3,5].

In the remainder 20% of cases, AMLs occur in association with TSC or, less commonly, as part of LAM. Relative to the sporadic form of AML, these hereditary lesions affect both genders equally and manifest at a younger age. They are likely to be multiple, large, and bilateral, and are prone to grow and be more aggressive [3,5,7].

AMLs are observed in approximately 75% of patients with TSC. TSC is an autosomal dominant multisystem disorder characterized by the development of benign tumours (hamartomas) in multiple organs throughout the body. The main organs involved are the brain, skin, lung, and kidney [8]. TSC is caused by mutations in one of two genes, TSC1 and TSC2, which encode the proteins hamartin and tuberin, respectively. These proteins interact with each other to form a tumour suppressor complex, which inhibits the mammalian target of rapamycin (mTOR) pathway. This pathway is important for angiogenesis, protein synthesis, and cell growth. Defective or deficient TSC1 or TSC2 activity leads to unchecked activation of mTOR and formation of characteristic hamartomas [2,6,7].

AMLs may also develop in patients with LAM. This rare disorder may occur sporadically, in the absence of other diseases, but is common in patients with TSC. It is characterized by diffuse interstitial proliferation of smooth muscle cells (LAM cells) and the presence of thin-walled cysts distributed throughout the lungs. These LAM cells have mutations in the same TSC1 and TSC2 genes [6,7].

### Histological Classification

Renal AML can be classified histologically as typical (triphasic) or atypical (monophasic or epithelioid) [5].

Most AMLs contain all three components, namely dilated blood vessels (angio), smooth muscle cells (myo), and mature adipocytes (lipo), in various proportions. They are classified as triphasic tumours. However, some tumours consist almost exclusively of one component, while other elements are present in very small amounts. They are called monophasic. At last, the epithelioid variant of AML contains numerous epithelioid muscle cells with abundant eosinophilic and granular cytoplasm and few or no fat cells. These tumours have a tendency toward malignant transformation and can be locally aggressive. Histologically, they can resemble and be misdiagnosed as RCC. However, distinction of epithelioid AMLs from RCC is possible by the presence of immunohistochemistry markers, such as smooth muscle markers (caldesmon and smooth muscle actin) and melanocytic markers (HMB-45 antigen and melan-A) [2,3,4,5].

### Radiological Classification

There is a growing body of literature describing findings and techniques that may be used to distinguish renal AMLs. A lack of a generally accepted and standardized terminology or classification of AML fuels the persisting confusion. In this review, a radiological classification reported by two groups of authors will be discussed.

A recent article hypothesized that renal AMLs can be classified according to CT and MRI findings using quantitative values. They classified these tumours as fat-rich, fat-poor, or fat- invisible, based on the amount of fat detected on imaging studies. To distinguish these different types, a region of interest (ROI) was placed in the most hypodense area of the lesion to measure the attenuation value. A lesion was defined as fat-rich when the ROI measured ≤ –10 Hounsfield units (HU) on unenhanced CT (UECT). However, UECT could not differentiate fat-poor from fat-invisible AML. Therefore chemical shift imaging (CSI) MRI was introduced. This modality provides a higher sensitivity for fat detection than UECT. As in UECT, the most signal-dropped area was detected and a ROI was placed within. On CSI-MRI, fat-poor AML was characterized when the tumour-to-spleen ratio (TSR) was < 0.71 or the signal intensity index (SII) was > 16.5%. Because of too little fat, fat-invisible AML was defined having a TSR ≥ 0.71 and a SII ≤ 16.5% [9,10].

This classification showed an almost perfect inter-reader agreement, which means it is feasible for radiologists to apply in practice. However, it is important to understand that detection of the most hypodense area on CT or most signal-dropped area on MRI and placement of the ROI within is crucial. A mislocation or inappropriate size of the ROI may change the type of AML, with misclassification and unwanted procedures as a result. Another disadvantage is the fact that different CT and MR scanners may produce different attenuation numbers and signal intensities, leading to a wrong classification. For example, if an AML is measured –9 HU at one CT scanner, it will be classified as fat-poor. However, if another scanner describes the lesion as –11 HU, it will be classified as fat-rich. Although the difference of 2 HU is very minimal and probably insignificant, the management is completely different [9,10].

Another review article proposed a classification based on clinical, histologic, and imaging features. First, the authors divided renal AMLs into sporadic and hereditary. Histologically, they classified sporadic AMLs into benign triphasic type and potentially malignant epithelioid type [3,4].

Benign triphasic type was further divided into fat-rich and fat-poor AML using imaging modalities. While fat-rich AML contained enough fat to be detected on UECT (ROI ≤ –10 HU), fat-poor AML did not. Fat-poor AML was further classified into three subtypes, based on the amount and distribution of adipocytes within the lesion and other imaging features: hyperattenuating AML, isoattenuating AML, and AML with epithelial cysts. Hyperattenuating AML was hyperattenuating compared to renal parenchyma on UECT (> 45 HU), homogeneously enhancing on contrast enhanced CT, and T2-hypointense on MRI. On the contrary, isoattenuating AML was characterized by attenuation numbers close to renal parenchyma on UECT (between –10 and 45 HU). These lower attenuation levels were caused by the presence of fat cells, sufficient in quantity to lower the overall attenuation relative to hyperattenuating AMLs, but too few in one area to be detected on regular imaging. Finally, AMLs with epithelial cysts could be distinguished by the presence of epithelial cysts, with the non-cystic parts appearing similar to hyperattenuating AML. Radiological features of this rare subtype could not be fully described, due to the lack of data [3,4].

Epithelioid AMLs, that can be potentially malignant and metastasize, showed hyperattenuation on UECT, heterogeneously enhancement on contrast enhanced CT, and T2-hypointensity on MRI. These findings were caused by the epithelioid muscle component and the intratumoral haemorrhage and necrosis [3,4,10].

There are limitations associated with the classification described above. First, the requirement of clinical and pathological information makes it difficult for radiologists to use in daily practice. Second, there are problems in applying the imaging criteria for classifying isoattenuating AML. The authors reported isoattenuating AML being an AML with attenuation values between –10 and 45 HU on UECT. However, simple renal cysts have attenuation numbers ranging between –10 and 10 and appear definitely hypoattenuating, while renal masses measuring > 40 HU frequently appear hyperattenuating. So, because of the wide range of AML attenuation values, “isoattenuating AML” does not seem to be an appropriate term. Furthermore, the authors did not suggest a quantitative threshold for CSI-MRI, which means that radiologists cannot classify AML using TSR or SII [9].

In conclusion, the classification first described is the most easily applicable in practice. Radiologists can categorize renal AMLs as fat-rich, fat-poor, and fat-invisible, according to the amount of fat detected on UECT or CSI-MRI. However, attention should be paid to fit the ROI within the right area of the fat tissue, otherwise many fat-rich or fat-poor AMLs may be misclassified as fat-poor or fat-invisible. Table 1 summarizes the features of this classification.

**Table 1:** Radiological classification of renal AML [9].

	UECT Region of interest (ROI)	MRI-CSI Tumour-to-spleen ratio (TSR)	MRI-CSI Signal intensity index (SII)

*Fat-rich AML*	≤ –10 HU	< 0.71	> 16.5%
*Fat-poor AML*	> –10 HU	< 0.71	> 16.5%
*Fat-invisible AML*	> –10 HU	≥ 0.71	≤ 16.5%

## Diagnosis

### Clinical Presentation

The increased use of cross-sectional imaging and advances in imaging technology explain that the majority (> 80%) of AMLs are now incidentally found. Most patients are asymptomatic when they receive the diagnosis of an AML [2].

Symptomatic presentation is most frequently related to spontaneous retroperitoneal haemorrhage, although this is seen in less than 15% of cases [2]. This may lead to shock in one-third of patients. Therefore, risk of a life-threatening bleeding is the main clinical concern in a patient diagnosed with AML. Other symptoms and signs include a palpable mass, flank pain, haematuria, anaemia, urinary tract infection, or renal failure [11,12].

In contrast to the benign prognosis of classic renal AMLs, the epithelioid variant may undergo malignant transformation, although this is rare. This is manifested by local aggressiveness, including lymphadenopathy, and distal metastases. The larger the tumour, the more likely it is to spread [3,11].

In TSC and LAM, the disease process tends to be more aggressive compared to the sporadic form. This implicates that symptomatic presentation is more common in this subgroup [6,12].

In TSC, hamartomas are formed in multiple organs throughout the body, leading to a variety of symptoms. About 90% of patients with TSC have skin manifestations, with a range from facial angiofibromas to hypomelanotic macules. Neurological symptoms, including epilepsy, behavioural problems, and cognitive impairment, occur in up to 85% of patients. Renal AMLs are present in approximately 75% of patients with TSC and manifest as bilateral and multiple tumours, with a high tendency to grow and cause spontaneous bleeding. LAM, in association with TSC or in the sporadic form, is characterised by cystic changes within the lung parenchyma. This may lead to chylous pleural effusions, recurrent pneumothoraces, and cystic lung disease [2,6,7,8].

### Imaging Findings

Typical renal AML can be diagnosed accurately based on imaging findings. The demonstration of macroscopic fat within a lesion is the hallmark feature on all modalities. However, AML represent a diagnostic challenge for every imaging method in case of haemorrhage, calcification, necrosis, or low fat content. All imaging features of renal AML are summarized in Table 2.

**Table 2:** Imaging features of renal AML [2,3,4,9,10,11].

	US	UECT	MRI-T1	MRI-T2	MRI-CSI

*Fat-rich AML*	Markedly hyperechoic	Hypoattenuating	Signal loss on FS	Hyperintense	Decrease in signal intensity
*Fat-poor AML*	Slightly hyperechoic	Heterogeneously isoattenuating or hyperattenuating	+/– signal loss on FS	Heterogeneously or homogeneosly hypointense	Decrease in signal intensity
*Fat- invisible AML*	Isoechoic	Homogeneously hyperattenuating	No signal loss on FS	Homogeneously hypointense	No decrease in signal intensity

*Note:* FS = fat suppression; +/– = loss of signal on fat-suppressed MRI may or may not be present.

#### Ultrasound

**Figure 1:**
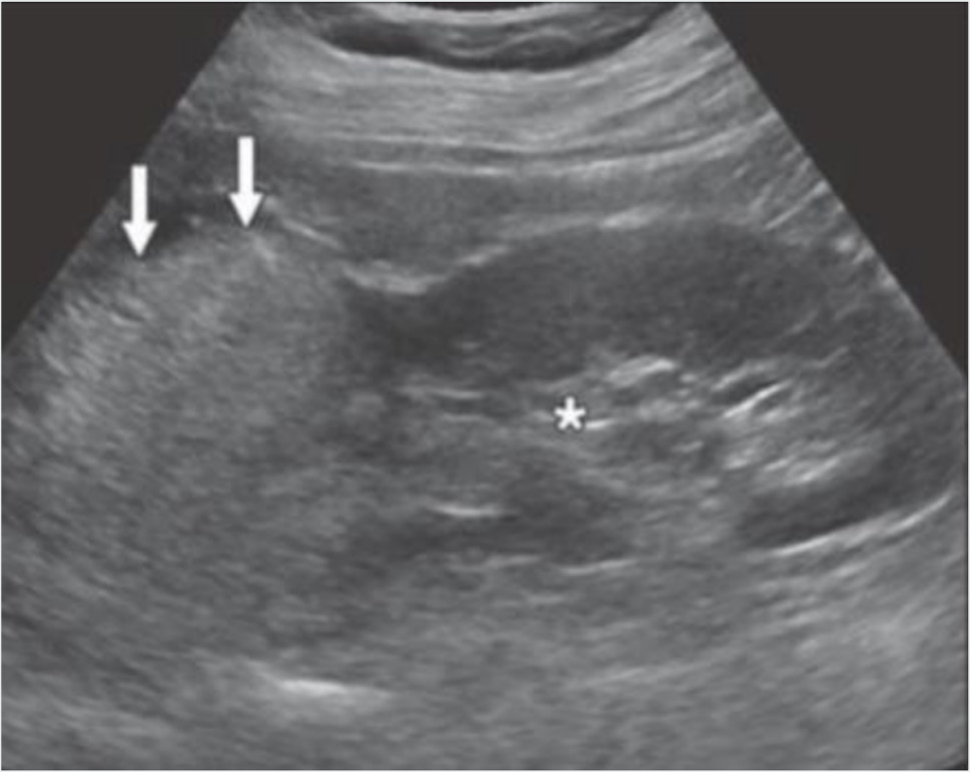
US image showing a fat-rich AML (arrow). The tumour is more hyperechoic than the right renal sinus (asterisk) [10].

The classical appearance of a fat-rich AML on ultrasound (US) is that of a hyperechoic lesion with a posterior acoustic shadow (Figure 1). The hyperechoic nature of a classic AML is the result of the combination of its fat, blood vessel, and muscle contents, while the shadowing is due to the multiple tissue interfaces between those different elements [11]. The echogenicity of the mass is the same as or greater than that of the renal sinus [2,3,4]. However, this hyperechogenicity is not a constant finding. As the amount of fat deceases, the echogenicity of the lesion decreases. Fat-poor AML has a mixed echotexture, being hyperechoic and isoechoic compared with renal parenchyma. Fat-invisible AML is homogenously isoechoic with respect to renal parenchyma. The echogenicity of both tumours is less than that of the renal sinus. It is challenging to differentiate them from other renal lesions such as RCC, which has an echogenicity that is also less than that of the renal sinus [3,4,10]. The lack of sensitivity of US at defining small renal masses implicates that the diagnostic reliability of this imaging modality is not high enough to allow it to be used for diagnosing renal AMLs in daily practice [2,11]. Additional imaging studies may be needed to confirm the diagnosis, i.e. the presence of fat.

#### Computed Tomography

**Figure 2:**
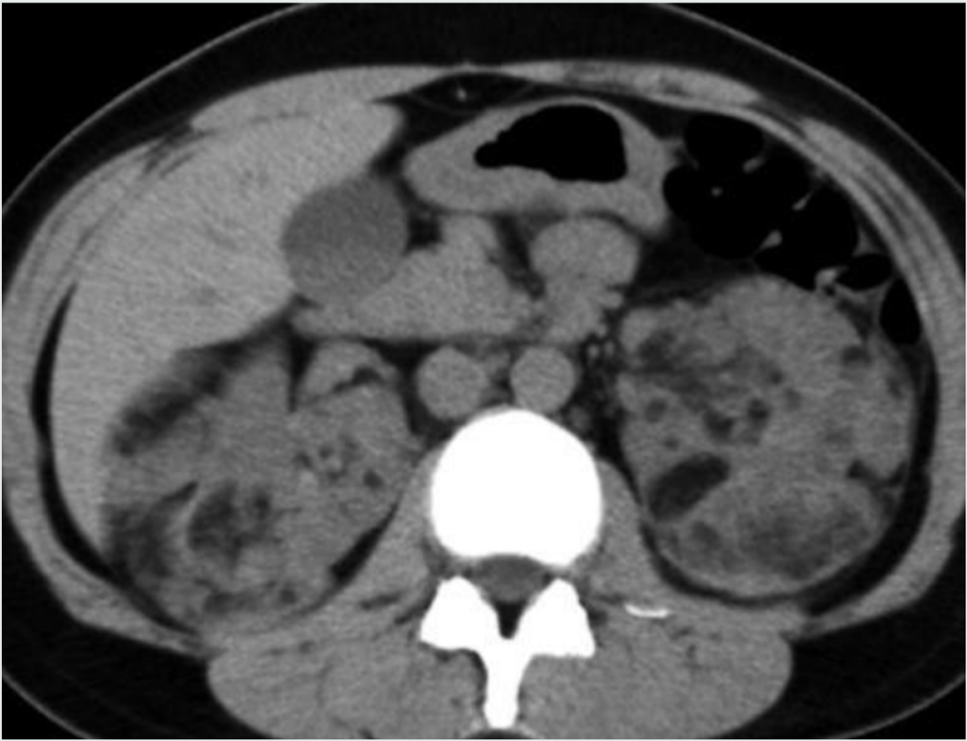
UECT showing bilateral and multiple AMLs in a patient with TSC. Each lesion contains attenuation numbers less than –10 HU, consistent with fat-rich AML [3].

**Figure 3:**
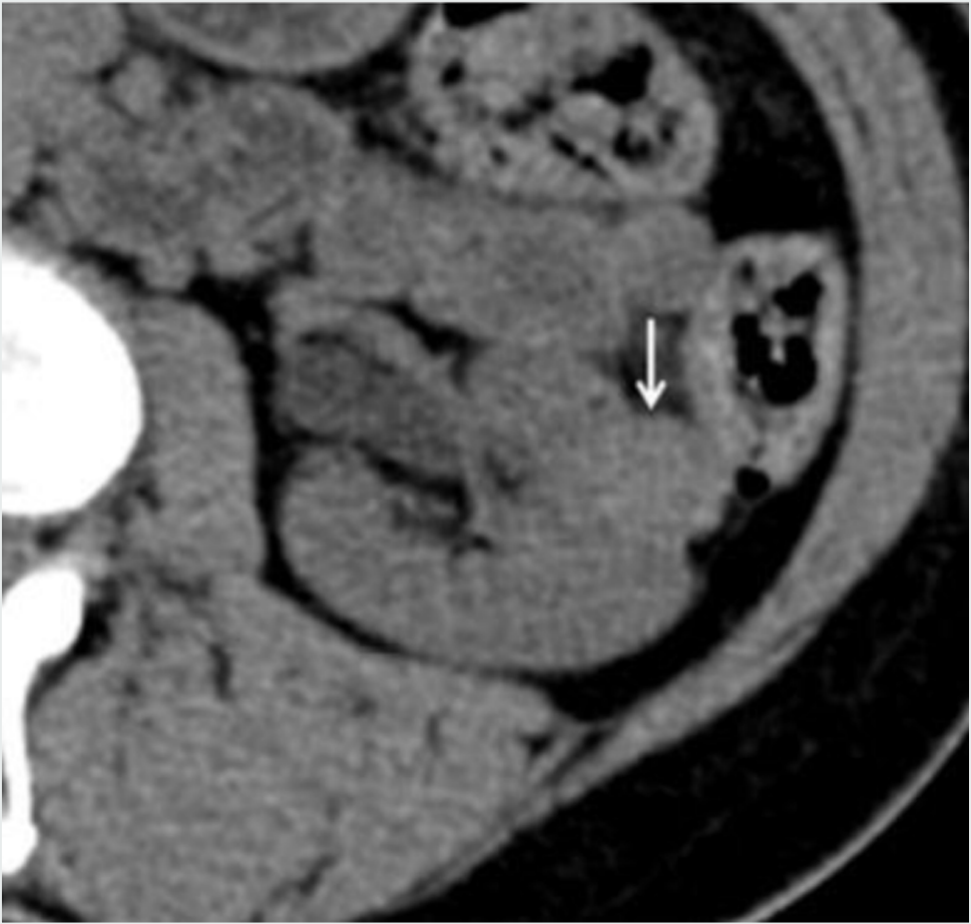
UECT demonstrating a left solid renal lesion (arrow). It appears homogeneously hyperdense and has attenuation values as high as 44 HU, consistent with fat-invisible AML [9].

The imaged-based detection of fat generally starts with CT. As on US, the characteristics of AMLs on UECT may vary due to variable quantities of angiogenic, myogenic, and fatty elements. The presence of ROIs containing attenuations of –10 HU or less is a reliable sign of an area of adipose tissue [3,4]. Therefore, detecting fat is feasible in most fat-rich AMLs (Figure 2). However, some fat-rich AMLs contain very small areas of fat, which may not be recognized at CT. Therefore, the acquisition of thin slice sections (1.5–3.0 mm) and obtaining attenuation measurements using small ROIs or even pixel values may be needed. Several authors reported a higher sensitivity for detecting small foci of fat when using pixel mapping, using a line or square of four pixels. In fat-poor AMLs, UECT cannot show a hypoattenuating area measuring less than –10 HU. The attenuation values of such lesions range widely, according to the size or location of a ROI: when a ROI is placed in a region of muscle cells and vessels, the lesion attenuation will be higher than when a ROI is placed in an area that consists mostly of fat cells. For this reason, fat-poor AMLs are heterogeneously isoattenuating or hyperattenuating. On the contrary, fat-invisible AMLs appear homogenously hyperattenuating (Figure 3). Because they contain too little fat cells, UECT is not able to show any fat attenuation in these lesions. So wherever a ROI is placed in fat-invisible AMLs, their attenuation numbers tend to be higher and fairly constant compared with those of fat-poor AMLs [9,10,11].

**Figure 4:**
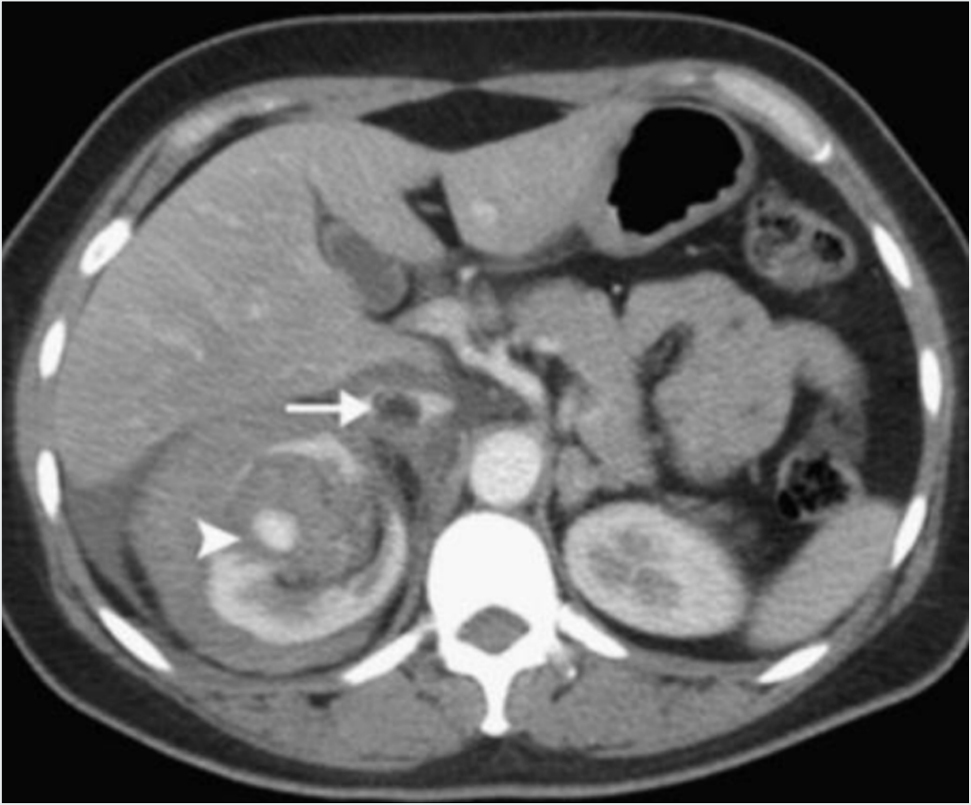
Contrast-enhanced CT showing two features: a perirenal hematoma and enhancing lesion with aneurysm formation (arrowhead) and a thrombus in the IVC (arrow) [15].

There are several other variants in which an AML can appear on UECT. Frequently, a perirenal or intratumoural haemorrhage is present (Figure 4). This hyperdense collection may obscure the fat, leading to misdiagnosing a simple AML as a renal cancer [2]. Lesions that contain fat and calcifications also represent a diagnostic challenge. As the presence of adipose tissue is highly suggestive of AML, the presence of calcification raises the possibility of renal cancer. However, several authors reported the existence of both AMLs with calcification and RCCs having fat without calcification [11,13,14]. Another, less frequent appearance is that of necrosis within a lesion, being rather typical for epithelioid AMLs [3,4].

Contrast-enhanced CT is not routinely indicated in diagnosing renal AMLs, but should be performed in selected cases. Frequently, large AMLs contain dilated blood vessels that have the potential of rupturing and bleeding. Whenever tumour bleeding is suspected, it is essential to identify the tortuous vessels using contrast-enhanced CT, before therapeutic embolization can be scheduled [10,15].

#### Magnetic Resonance Imaging

In the diagnosis of AML, MRI is equivalent in accuracy to CT. Several MRI sequences can be used. The classical approach is to locate fat within a mass by comparing T1-weighted images with and without frequency selective fat suppression. A classic fat-rich AML appears T1-hypointense with and T1-hyperintense without fat suppression [2,3,4]. However, this T1-hyperintensity is not a specific characteristic of AML and can also be present in RCC and haemorrhagic cysts. Moreover, fat-invisible AMLs contain too little fat to show any hypointensity on fat-suppressed T1-weighted images. In such cases, other techniques may help in differentiating between those entities [11,16].

**Figure 5:**
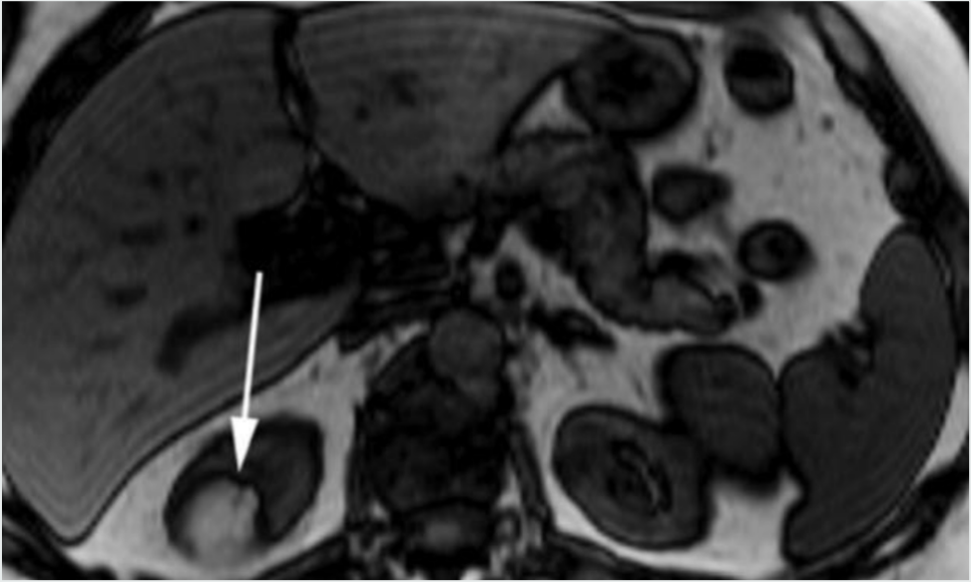
Opposed-phase T1-weighted MR image with a hyperintense renal mass. The India ink artefact (arrow) is present at the interface of the lesion with the kidney [16].

More recently, the application of MRI artefacts has been suggested to evaluate fat in renal tumours. One of them, the chemical shift artefact, is due to the differences between resonance frequencies of fat and water. This results in alternating high and low signals in the frequency encoding direction on opposed-phase imaging. This out-of-phase cancellation effect between fat and water gives rise to another MRI artefact, called the black boundary or India ink artefact. This artificially created black line is located at the junction of fat (present in AML cells) and water (present in renal parenchyma) and results in a sharp delineation of the muscle-fat boundary (Figure 5). This sign is indicative of an AML [3,4,11,16]. It is especially useful in fat-poor AMLs and very small lesions, in which the typical T1 features may not be noticeable [2]. However, fat-invisible AMLs do not show this decrease in signal intensity, because they contain too little fat cells [10].

T2-weighted images can also be used for diagnosing AML. Fat-rich AML typically is hyperintense with respect to renal parenchyma. It has hyperintense foci within a hypointense background, because areas of fat are distributed among blood vessels and muscle cells. Fat-poor AML is either homogeneous or heterogeneous hypointense compared with renal tissue, depending on the distribution of its areas of fat. If these areas are small but diffuse, the signal intensity is homogeneous, while it is heterogeneous when fat cells are located focal. Finally, fat-invisible AML is homogeneously hypointense in comparison to renal parenchyma on T2, because of the dominance of the muscle component and the paucity of adipose tissue [2,3,4,10].

Contrast-enhanced MRI has a limited role in the diagnosis of AML. After intravenous administration of gadolinium, renal AMLs show hypointense compared to renal parenchyma. However, these enhancement characteristics appear to be similar to those of hypovascular RCCs [11,17].

### Percutaneous Biopsy

Most AMLs can be diagnosed with imaging by identifying intralesional fat. However, in select cases, the use of imaging alone is insufficient and percutaneous renal biopsy may be necessary for correctly diagnosing the renal mass, thus avoiding unnecessary treatment. Nowadays, percutaneous biopsy is increasingly used to differentiate between benign and malignant renal lesions [18,19].

Currently, percutaneous renal biopsy is recommended only for differentiating fat-invisible AML from RCC, if both CT and MR images are inconclusive. A fat-invisible AML typically appears as a hyperattenuating mass that enhance homogenously on UECT. Although this presentation is uncommon for a RCC, both pathologies may appear identical on imaging. In these cases, the mass should be evaluated with MRI. While a fat-invisible AML is homogenously hypointense on T2-weighted MR images, clear cell RCC appears hyperintense [9]. However, the papillary subtype of RCC appears also hypointense. Therefore, percutaneous biopsy is required to differentiate fat-invisible AML and papillary RCC when a small (less than 3 cm), T2-hypointense renal mass without evidence of intratumoural fat is encountered [18]. If the lesion is larger (more than 3 cm) or if there is evidence of haemorrhage, percutaneous biopsy may be skipped. Proceeding directly to a suitable treatment may be a more appropriate option, both to avoid further bleeding and because epithelioid AML and RCC are more presumable [3].

### Differential Diagnosis

Over the last decades, the detection of small renal masses definitely increased by the increased use of cross-sectional imaging studies. A lot of these incidentally found renal lesions are usually presumed to be RCC and are treated as such. However, a recent study showed that 21.5% of those lesions turned out to be benign after surgery. AML accounted for nearly half of this group of benign masses. This shows that the differentiation between AML and RCC remains difficult in routine practice [20,21,22].

As already repeatedly stated above, classic AML can be distinguished from RCC by the presence of macroscopic fat in sufficient quantity to be detected on UECT. However, in 4–5% of AML cases, no fat can be visualized on CT, increasing the difficulty in differentiating it from RCC. Correctly diagnosing this fat-poor and fat-invisible AML requires sufficient clinical and imaging information. Clinical information supporting a diagnosis of AML includes a younger age, female sex, and asymptomatic presentation. Imaging findings that should raise the suspicion of AML include the absence of calcification, the absence of perinephric collateral vessels, multiple lesions, hyperattenuation in comparison to renal parenchyma on UECT, and hypointensity on T2-weighted MRI. However, because these characteristic features are not present in every AML and exceptions do exist, no single finding or modality is perfectly accurate [20,21,22,23,24].

## Treatment

As most renal AMLs are diagnosed incidentally in asymptomatic patients, therapeutic interventions are required in a minority of patients. Potential interventions include selective renal artery embolization, nephron-sparing surgery, complete nephrectomy, cryo- and radiofrequency ablation, and treatment with mTOR inhibitors.

### Indications

Historically, the main indications for intervention have been the presence of symptoms, the presence in women of childbearing age, suspicion of malignancy, and size larger than 4 cm. The use of a 4 cm tumour size as a criterion for treatment comes from a frequently quoted review published in 1986 [2,11,19]. The authors of this review reported that 82% of patients with renal AMLs larger than 4 cm experienced symptoms and 51% of them presented with active retroperitoneal haemorrhage. Other early series showed that patients with tumours larger than 4 cm had interval growth and needed treatment more often. They all suggested 4 cm as the limit above which an intervention should be considered. This threshold has been widely adopted for many years, although the understanding of the biology and management of the disease have changed significantly since then [25,26,27].

However, this threshold has recently been questioned. Recent studies do not support the 4 cm size criterion. One author found that only 30% of AMLs larger than 4 cm were symptomatic [2]. Another one showed that using a cut-off of 4 cm as predictor of haemorrhage has a lower specificity than an aneurysm size of 5 mm. In other series, the angiographic appearance of AMLs was used for analysing them. They found that lesions with high vascularity (multiple, large, tortuous vessels) were more likely to require intervention for bleeding. All this evidence would suggest that, although tumour diameter is important, the size of related aneurysms and the vascularity of the AML may ultimately be more significant [11,27].

As most AMLs now are found incidentally, physicians are challenged by a treatment dilemma when an AML in an asymptomatic patient reaches the 4-cm threshold. They are worried that these “large” lesions may rupture and cause life-threatening bleeding. Consequently, AMLs are often imaged intensively and repeatedly, and treated empirically once they attain a diameter of 4 cm [26].

The current guidelines of the European Association of Urology recommend intervention in well-selected cases, including symptomatic tumours, large lesions, presence in women of childbearing age, and poor access to follow-up or emergency care. A size threshold for treatment, however, remains controversial [2,24,25,26].

### Active Surveillance

Once the initial diagnostic evaluations are completed and indications for treatment are not present, active surveillance should be used to monitor progression of known tumours and development of new ones [29]. For sporadic AML, there are currently no guidelines on the frequency of imaging studies neither on which modality should be used. These decisions are likely to be institution dependent and should be guided by the individual clinical scenario [2]. Annual repeat of imaging seems to be appropriate for small, solitary lesions. For hereditary AML, the International Tuberous Sclerosis Complex Consensus recommends the use of MRI, because of its increased sensitivity in the detection of adipose tissue. Annual clinical evaluation of renal function and blood pressure is also required in these cases [19,28,30].

### Embolization

Historically, there was a greater tendency towards surgery in the treatment of renal AMLs. They were often excised because malignancy could not be excluded. However, this has shifted, as AMLs can now be confidently recognized at imaging. Selective transarterial embolization is now the first-line treatment option, especially in the event of acute bleeding or hemodynamic instability [2,5]. Many clinicians favour embolization and reserve surgery for patients with uncontrollable symptoms, vascular malformations, failure of embolization, and rare diagnostic uncertainty. When surgery needs to be performed, preoperative embolization of the lesion may be taken into consideration in reducing the difficulty and complications of tumourectomy or nephrectomy [11,19,27,32].

Embolization is associated with a relatively high percentage of side effects (42.8%), although most of these are post-embolization syndromes. This self-limiting condition is characterized by fever, flank pain, leucocytosis, nausea, and vomiting within the first three days after the procedure, and it is managed conservatively with standard supportive care. Routine prophylaxis, including antipyretics, antiemetics and analgesia, may play a role in periprocedural management [2,27]. Other complications, including non-target embolization of normal parenchyma or renal infarction with abscess forming, are uncommon [11,19,32]. In general though, arterial embolization is a well-tolerated procedure.

Although it is now seen as the first-line treatment for patients with AMLs, both regrowth and repeated haemorrhage after embolization remain a concern. The effect of embolization may vary, because of the varying amounts of adipose, vascular, and smooth muscle tissue in the lesions. Different authors showed in their series a need for reembolization varying from 17% till 37% [32,33,34].

### Surgery

Surgical excision in the form of partial or radical nephrectomy is the only treatment that completely removes the renal mass, although recurrence from other parts of the kidney may occur. Every surgery should rely on a nephron sparing approach whenever possible [2,5]. Parenchymal preservation is even more important in patients with TSC or LAM, because of the multifocal disease pattern and the higher recurrence rate. Nephrectomy is indicated only when a renal AML is very large, when suspicion of malignancy is high, and when other treatment options cannot be performed. However, in an emergency setting, a nephrectomy can be lifesaving [31,32].

### Ablation

When comparing current treatment methods, cryoablation and percutaneous radiofrequency ablation appear to be attractive alternatives to embolization or surgery. Different series demonstrate good efficacy with minimal complications, few repeat treatments, and no recurrences. However, reports of the use of these minimal invasive techniques are confined to small and asymptomatic lesions [19]. Little evidence is available on the application in larger or symptomatic tumours. Overall, ablation has shown some promise in the treatment of a specific AML group that some would say should be managed by active surveillance [2,33,34].

### Drugs

The identification of mutations in TSC1 and TSC2 and the existence of a licensed drug targeting the mTOR pathway, lead to rapid translation of mTOR inhibitors to patients with TSC and LAM. These medicines interrupt further tumour progression and promote reversion of existing lesions. Sirolimus, also known as rapamycin, was the first mTOR inhibitor analysed in treating hereditary AML. It was originally developed as immunosuppressant for use in organ transplantation. Overall, sirolimus was well tolerated in these early studies, with mouth ulcers, skin lesions, dyslipidaemia, and proteinuria as most commonly encountered side effects. It is now approved for treatment of LAM [6]. Everolimus, another mTOR inhibitor and rapamycine derivative, has been studied the most. Currently, the Food and Drug Administration and European Medicines Agency approve this agent for treating AML in the setting of TSC [32,35,36]. The position of mTOR inhibitors in the management of sporadic AML still remains to be determined [2].

For asymptomatic AMLs in TSC and LAM that are enlarging and larger than 3 cm, recent guidelines suggest that mTOR inhibition currently is the most effective first-line treatment. The demonstrated tolerability so far to date is superior to the renal impairment caused by AML progression and is preferable to other treatment modalities [28]. Another concern for treating physicians is how long the treatment with those drugs should continue, as the effect of mTOR inhibition is reversible. To continue the treatment for AML, consideration should be given to long-term complications, cost, and safety, compared with other treatment options [32,36].

## Conclusion

Renal AML is the most prevalent benign neoplasm of the kidney. It has a variable and heterogeneous nature, with the potential to pose serious diagnostic challenges in clinical practice. The characteristics of classic AMLs are well described, but the radiological distinction between non-classic AMLs and RCCs continues to be difficult. Knowledge of the different types, their classification, and their radiologic appearance will help radiologists in making a correct diagnosis. When an AML is considered in the differential diagnosis of a renal lesion that does not demonstrate classical features, confirmatory imaging, percutaneous renal biopsy, or surgical excision should be performed. Several invasive, non-invasive, and pharmacologic treatment options exist. Careful surveillance before and after treatment is necessary, particularly for patients with TSC or LAM. Without proper management, renal AML may have serious consequences.
